# Evaluation of an ARF diagnosis calculator: a survey and content analysis

**DOI:** 10.1186/s12911-022-01816-7

**Published:** 2022-03-26

**Authors:** Elizabeth Fisher, Christian James, Diana Mosca, Bart J. Currie, Anna P. Ralph

**Affiliations:** 1 Division of Medicine, Royal Darwin Hospital, Darwin, NT Australia; 2 RHD Australia, Darwin, Australia; 3 Global and Tropical Health, Menzies School of Health Research, Charles Darwin University, Darwin, NT Australia

**Keywords:** Acute rheumatic fever, Rheumatic heart disease, mHealth, Clinical practice guidelines, Indigenous Australian Health

## Abstract

**Background:**

Acute Rheumatic Fever (ARF) is a critically important condition for which there is no diagnostic test. Diagnosis requires the use of a set of criteria comprising clinical, laboratory, electrocardiographic and echocardiographic findings. The complexity of the algorithm and the fact that clinicians lack familiarity with ARF, make ARF diagnosis ideally suited to an electronic decision support tool. The *ARF Diagnosis Calculator* was developed to assist clinicians in diagnosing ARF and correctly assign categories of ‘possible, ‘probable’ or ‘definite’ ARF. This research aimed to evaluate the acceptability, accuracy, and test performance of the *ARF Diagnosis Calculator.*

**Methods:**

Three strategies were used to provide triangulation of data. Users of the calculator employed at Top End Health Service, Northern Territory, Australia were invited to participate in an online survey*,* and clinicians with ARF expertise were invited to participate in semi-structured interviews. Qualitative data were analysed using inductive analysis. Performance of the calculator in correctly diagnosing ARF was assessed using clinical data from 35 patients presenting with suspected ARF. Diagnoses obtained from the calculator were compared using the Kappa statistic with those obtained from a panel of expert clinicians.

**Results:**

Survey responses were available from 23 Top End Health Service medical practitioners, and interview data were available from five expert clinicians. Using a 6-point Likert scale, participants highly recommended the *ARF Diagnosis Calculator* (median 6, IQR 1), found it easy to use (median 5, IQR 1) and believed the calculator helped them diagnose ARF (median 5, IQR 1). Clinicians with ARF expertise noted that electronic decision making is not a substitute for clinical experience. There was high agreement between the *ARF Diagnosis Calculator* and the ‘gold standard’ ARF diagnostic process (κ = 0.767, 95% CI: 0.568–0.967). Incorrect assignment of diagnosis occurred in 4/35 (11%) patients highlighting the greater accuracy of expert clinical input for ambiguous presentations. Sixteen changes were incorporated into a revised version of the calculator.

**Conclusions:**

The *ARF Diagnosis Calculator* is an easy-to-use, accessible tool, but it does not replace clinical expertise. The calculator performed well amongst clinicians and is an acceptable tool for use within the clinical setting with a high level of accuracy in comparison to the gold standard diagnostic process. Effective resources to support clinicians are critically important for improving the quality of care of ARF.

**Supplementary Information:**

The online version contains supplementary material available at 10.1186/s12911-022-01816-7.

## Background

Acute Rheumatic Fever (ARF) is an autoimmune condition of childhood triggered by infection with the bacterium Group A Streptococcus. The highest documented rates occur in Aboriginal populations in Australia’s Northern Territory [1]. ARF causes significant morbidity, but most concerningly, leads to rheumatic heart disease (RHD), a valvular heart condition which only develops after severe or repeated ARF episodes, with consequent high complications including premature death [2].

**Fig. 1:**
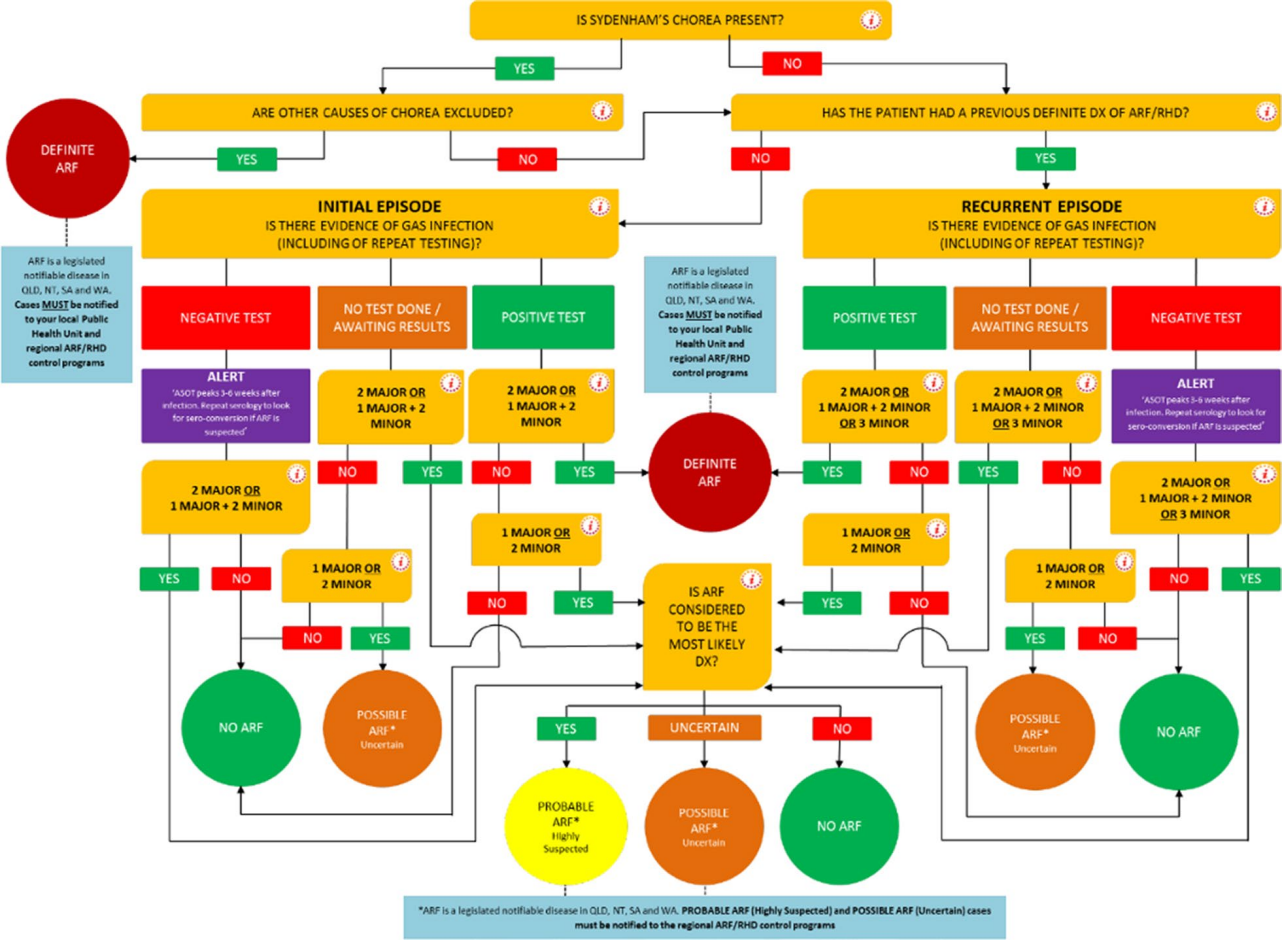
Algorithm for diagnosing acute rheumatic fever as available in 2016, illustrating the complexity of a paper-based algorithmic approach. Modified from Remond 2014 [6]

Timely diagnosis of ARF is critically important to ensure that secondary prevention with regular, long term antibiotic prophylaxis is commenced [3]. An ARF diagnosis also provides an important opportunity to provide culturally appropriate education for patients and their families about ARF prevention. However, there is no diagnostic test for ARF. Instead, diagnosis requires an experienced clinician to work through a set of criteria, the Jones Criteria, comprising clinical, laboratory, electrocardiographic and echocardiographic findings [4]. The Jones criteria (Table 1) have been revised on multiple occasions since first developed in 1944 and need to be applied slightly differently in first and recurrent episodes, and in low- and high-risk epidemiological settings, resulting in a complex algorithm (Fig. 1) [5].

**Table 1:** Updated Australian criteria for ARF diagnosis

	High-risk groups^†^	Low-risk groups
Definite initial episode of ARF	2 major manifestations + evidence of preceding Strep A infection, OR 1 major + 2 minor manifestations + evidence of preceding Strep A infection	
Definite recurrent episode of ARF in a patient with a documented history of ARF or RHD	2 major manifestations + evidence of preceding Strep A infection, OR 1 major + 2 minor manifestations + evidence of preceding Strep A infection, OR 3 minor manifestations + evidence of a preceding Strep A infection	
Probable or possible ARF (first episode or recurrence)	A clinical presentation in which ARF is considered a likely diagnosis but falls short in meeting the criteria by either: • One major or one minor manifestation, OR • No evidence of preceding Strep A infection (streptococcal titres within normal limits or titres not measured) Such cases should be further categorised according to the level of confidence with which the diagnosis is made: • Probable ARF (previously termed ‘probable: highly suspected’) • Possible ARF (previously termed ‘probable: uncertain’)	
Major manifestations	Carditis (including subclinical evidence of rheumatic valvulitis on echocardiogram) Polyarthritis or aseptic monoarthritis or polyarthralgia Sydenham chorea Erythema marginatum Subcutaneous nodules	Carditis (including subclinical evidence of rheumatic valvulitis on echocardiogram) Polyarthritis Sydenham chorea Erythema marginatum Subcutaneous nodules
Minor Manifestations	Fever ≥ 38 °C Monoarthralgia ESR ≥ 30 mm/h or CRP ≥ 30 mg/L Prolonged P-R interval on ECG	Fever ≥ 38.5 °C Polyarthralgia or aseptic monoarthritis ESR ≥ 60 mm/h or CRP ≥ 30 mg/L Prolonged P-R interval on ECG

^†^High-risk groups are those living in communities with high rates of ARF (incidence > 30/100,000 per year in 5–14-year-olds) or RHD (all-age prevalence > 2/1000). Aboriginal and Torres Strait Islander peoples living in rural or remote settings are known to be at high risk. Data are not available for other populations but Aboriginal and Torres Strait Islander peoples living in urban settings, Māori and Pacific Islanders, and potentially immigrants from developing countries, may also be at high risk

*CRP* C-reactive protein, *ECG* electrocardiogram, *ESR* erythrocyte sedimentation rate

Healthcare providers, especially those new to ARF-endemic settings (where staff turnover is often very high) can lack familiarity with ARF and therefore fail to recognise it, since ARF is now extremely rare in affluent settings due to the success of modern public health measures [7, 8]. As a result, ARF is under-diagnosed. In Australia’s Northern Territory, 75% of RHD cases lacked a prior ARF diagnosis, meaning the preceding ARF episodes(s), and opportunities for secondary prevention, had been missed [9].

Mobile health (mHealth) interventions have been rapidly increasing, with increasing access to technology, to assist clinicians with diagnostic and management decisions. In emergency departments in Canada, the provision of clinical decision rules via an app called “The Ottawa Rules” was found to be highly supported by clinicians and led to an expansion to include decision support for CT investigations, transient ischaemic attacks, and subarachnoid haemorrhages [10, 11]. Evaluations indicated a strong request from clinicians for further expansions. Similarly, a mobile app that assisted with the diagnosis of gestational diabetes mellitus was evaluated and identified consistent diagnostic accuracy and usability amongst 22 participants, advocating for its use as a clinical adjunct to diagnosis [12]. Both apps provided easy to access guidelines and decision support, bridging the barriers involved in paper-based complex algorithms within medicine.

ARF diagnosis and management are therefore ideally suited to ‘mHealth’ in the form of an electronic decision support tool [13]. RHDAustralia, an Australian Government funded agency that creates and disseminates ARF and RHD resources for clinicians and patients and their families, produces full-length, evidence-based, expert national guidelines and a summarised version, and has abridged these further for electronic format as a smart device application [3, 7]. The app (‘RHDApp’) includes an *ARF Diagnosis Calculator* for use by clinicians in diagnosing ARF. This can be used instead of referring to the table or the pictorial algorithm which is excessively complex (Table 1, Fig. 1). The *ARF Diagnosis Calculator* was first developed in 2014 and has been updated as national or international guidance has evolved. This research aimed to evaluate the acceptability, accuracy, and test performance of the *ARF Diagnosis Calculator.*

## Methods

### Setting

The study was undertaken in 2019–2020 at Top End Health Service, Northern Territory, Australia. Top End Health Services governs Royal Darwin Hospital, a tertiary referral hospital, as well as regional hospitals and primary care facilities in northern Australia where rates of ARF are high.

Approval for the study and research protocols were granted from the Human Research Ethics Committee (HREC) of the Northern Territory Department of Health and Menzies School of Health Research (HREC-19-3505 and HREC-18-3126). All methods were conducted in accordance with ethical guidelines and regulations. Informed consent was collected from all participants.

The following methods describe design and development of the ARF Diagnosis Calculator, and evaluation using triangulating approaches: a user survey, user interviews and comparison to a ‘gold standard’ diagnostic process.

### Design

This is a prospective study in which end-users of the RHDApp and the embedded *ARF Diagnosis Calculator* were surveyed and clinicians with expertise in ARF were interviewed. Additionally, we assessed performance of the calculator for correctly assigning a diagnosis of possible, probable or definite ARF, or not ARF, applied to Royal Darwin Hospital patients, compared with a ‘gold standard’ ARF diagnostic tool comprising a panel of expert clinicians. These three strategies (survey, interviews and comparison of diagnosis outcomes) were used to provide triangulation of data and gain broad insights into the tool. Finally, findings were shared with developers of the *ARF Diagnosis Calculator* and changes were incorporated to create a revised version.

### Development of the *ARF Diagnosis Calculator*

**Fig. 2:**
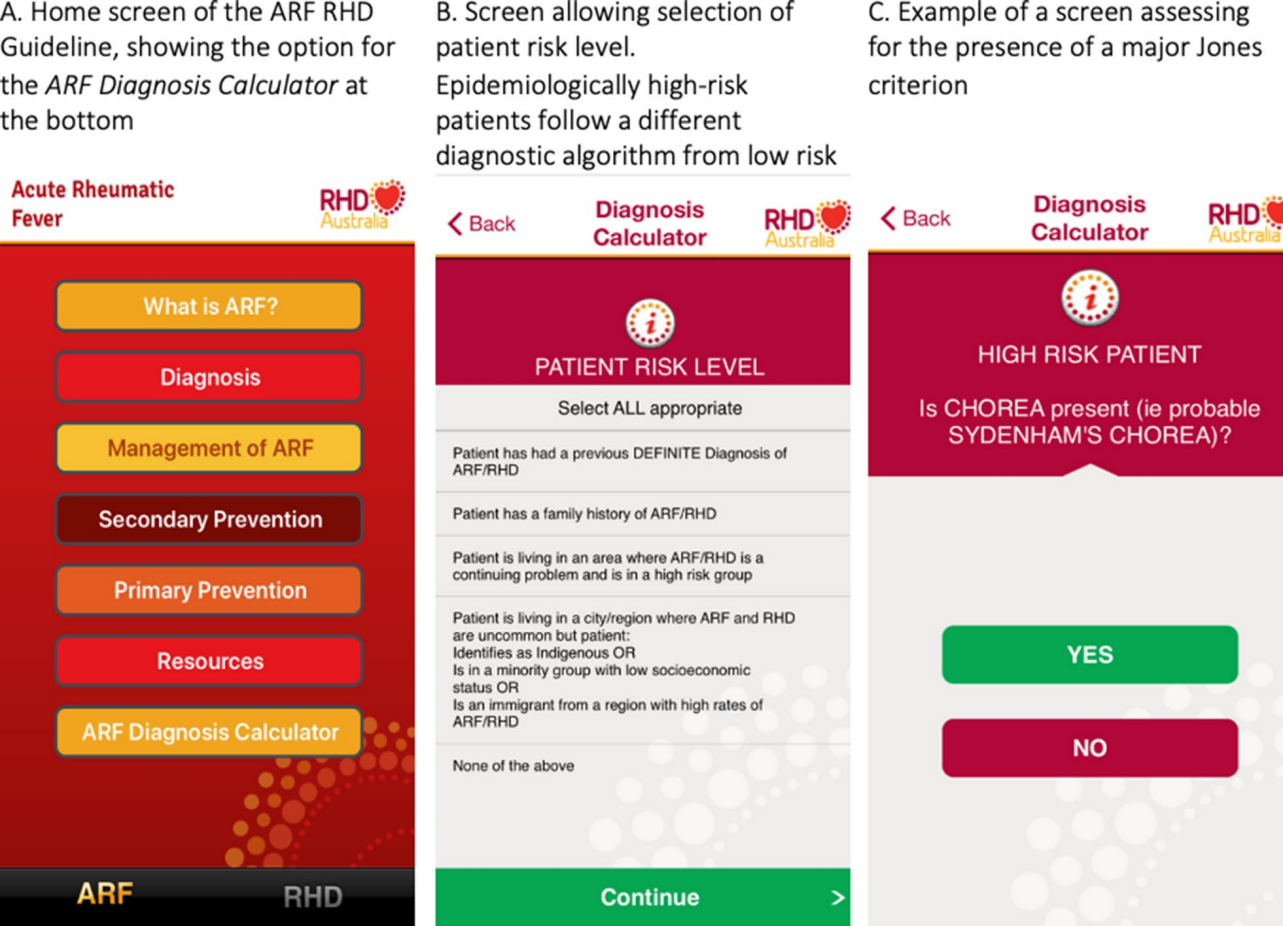
2020 ARF diagnosis calculator [14]

The prototype *ARF Diagnosis Calculator* was developed by RHDAustralia in 2014 within a condensed app version of the Australian ARF-RHD Guideline (RHDApp). The goal was to create an intuitive, simple tool to minimise ARF diagnostic complexities and assist clinicians in diagnosing ARF. The calculator was tested internally by nurses and doctors associated with RHDAustralia, then subjected to beta testing by Top End Health Service clinicians from a range of specialities and levels of seniority. Feedback to improve functionality of the *ARF Diagnosis Calculator* was received including suggestions to use traffic light-style colour coding, changing wording in message boxes for improved clarity, and identifying ambiguities or mistakes in the algorithm which resulted in incorrect or ambiguous assignment of diagnosis. The revised version was released in 2014, and further updated in 2015. The RHDApp has been downloaded more than 13,000 times in 13 countries on both Apple and Android platforms. It is available free of change, identified by searching for ‘ARF RHD Guideline’ in smart device app stores. The appearance of the RHDApp and the embedded *ARF Diagnosis Calculator* at the time of evaluation are provided in Fig. 2.

### Survey data collection and analysis

First, medical professionals were invited to participate in an online survey about their experience of using the RHDApp and the embedded *ARF Diagnosis Calculator*. A 30-question survey was developed using *Qualtrics*^*XM*^ software and distributed via email to medical professionals employed by Top End Health Service (comprising several hundred recipients across primary to tertiary care). The invitation email explained that the survey was open to registered or provisionally registered medical professionals who had the RHDApp available on their device and had used the *ARF Diagnosis Calculator*, and that participation was an indication of consent for use of the data provided for research purposes. The HREC approval number was provided in the cover email. There were no incentives provided to complete the survey. Repeat emails were sent as prompts to undertake the survey. The survey contained multiple choice questions, 6-point Likert scales (1: strongly disagree, 2: disagree, 3: slightly disagree, 4: slightly agree, 5: agree, 6: strongly agree) and free text entry opportunities to identify participant demographics, perceptions on usability of the overall RHDApp and the *ARF Diagnosis Calculator*, acceptability and physician confidence in the calculator. Responses to Likert scale questions were summarised as median and interquartile ranges (Table 2).

**Table 2:** Survey analysis

Question	Median	IQR
The *ARF Diagnosis Calculator* is easy to use	5	1
Using the *ARF Diagnosis Calculator* is easier than using the hardcopy guidelines	6	1
The *ARF Diagnosis Calculator* is accessible and quick to use when evaluating a patient	5	1
Using the *ARF Diagnosis Calculator* is laborious during a clinical encounter	2	1
I would prefer to use hardcopy guidelines	2	0.5
Using the *ARF Diagnosis Calculator* on my personal mobile device helps me to identify possible, probable and/or definite cases of acute rheumatic fever, or to exclude a diagnosis of ARF	5	1
The *ARF Diagnosis Calculator* risk assessment helps me to categorise my patients into low-risk and high-risk settings	5	0
I am confident with the results that I get from using the *ARF Diagnosis Calculator*	5	0
The ARF Diagnosis calculator improves my knowledge regarding acute rheumatic fever	5	1
The ARF Diagnosis calculator assists me in determining appropriate treatment plans for patients with possible, probable and/or definite ARF	5	0
I am more confident in the decisions that I make regarding an ARF diagnosis with the ARF Diagnosis calculator	5	0
I would recommend the ARF diagnosis calculator to my colleagues	6	1

### Interview data collection and analysis

Second, clinical specialists with expertise in ARF and RHD were invited to participate in semi-structured interviews of approximately 15 min. Purposive sampling of staff at Royal Darwin Hospital was used to engage different speciality physicians (General Medicine, Infectious Diseases, Cardiology) in adult and paediatric medicine with experience in ARF/RHD. Physicians who had active roles in clinical management, clinical research and/or policy development relating to ARF and RHD were eligible provided they were not affiliated with RHDAustralia or involved in development of the RHDApp. Potential participants were not excluded if they had participated in the survey. We aimed to enrol five individuals. Consenting participants were provided with dummy cases prior to the interview to reaccustom themselves with the *ARF Diagnosis Calculator*. An interview guide was developed to assess the usability, accuracy and physician confidence in the use of the calculator. Interviews were undertaken by a medical officer (E.F.) employed by Top End Health Service, who was not involved in development or promotion of the RHDApp. Interviews were audio-recorded and transcribed verbatim. Recordings were deidentified and coded using *NVivo* software, initially via open coding, identifying important information relevant to the research questions. Data were then analysed using an inductive content analysis approach within a constructivist epistemological framework. The inductive content analysis used codes, or frequently used terms, identified through interviews to generate a coding tree that summated all five interviews. Themes related to the research questions were then derived from this tree. Underpinning this content analysis was a constructivist epistemological framework which involves the emergence of theories grounded in the human experience of the interview subject, considering context and perception [15].

### Diagnostic performance based on patient presentations

The third evaluation strategy comprised comparison of performance of the *ARF Diagnosis Calculator* with a ‘gold standard’ diagnostic strategy. Patients presenting to Royal Darwin Hospital with suspected ARF were consecutively enrolled into a descriptive study (The START study (‘Searching for a Technology-Driven Acute Rheumatic Fever Test’), HREC-18-3126, 2018-ongoing) [16]. For each consenting participant, a team of clinical experts determined a final diagnosis of possible, probable or definite ARF, or not ARF, on the basis of all available diagnostic information including history, examination findings, laboratory results, electrocardiogram, echocardiogram and any other relevant imaging. This was considered the ‘gold standard’ diagnosis. The panel included author A.P.R and at least two other clinicians. To compare the performance of the *ARF Diagnosis Calculator*, summarised raw patient data for 35 cases with diagnoses already assigned by the panel, were presented to a practitioner blinded to the panel’s diagnosis (E.F.) to be entered into the *ARF Diagnosis Calculator*. In cases where the calculator sought a clinical decision i.e. “Is ARF considered to be the most likely diagnosis?” for consistency, the answer was completed as “uncertain”. Measurement of agreement between the clinical panel’s diagnosis and the calculator’s diagnosis was calculated using the Kappa statistic. Where diagnoses differed, the investigator identified the steps in the algorithm in which alternative choices might have been entered given the clinical data provided in order to determine potential areas of ambiguity within the *ARF Diagnosis Calculator*.

## Results

### Survey

35 doctors participated in the survey. Two commenced but did not complete the survey, 10 were excluded as they had not downloaded the RHDApp or had not used it, providing 23 completed surveys for analysis.

Of those using the *ARF Diagnosis Calculator* 57% were 25–34 years, 26% were 35–44 years and 17% were 45–54 years of age. Participants were of differing levels of seniority with 4% being interns, 22% residents, 39% registrars and 35% consultants. Clinicians had varying levels of experience in the Top End Health Service, with 39% of clinicians having worked for 2–5 years in this service. Respondents were predominantly working within outer regional Australia (35%) with 17% working in very remote and remote areas of Australia. A third of respondents indicated they would see a potential case of ARF once a week and another third estimated seeing a case once every 3–6 months. 70% of respondents said they were somewhat confident in diagnosing a case of possible, probable or definite ARF.

Survey responses are shown in Table 2 with median and interquartile ranges. The *ARF Diagnosis Calculator* was highly recommended (Median 6 out of 6, IQR 1). Participants considered it easy to use (Median 5, IQR 1) and strongly agreed that it was easier to use than hardcopy guidelines (Median 6, IQR 1). Participants were confident in the results obtained from the calculator (Median 5, IQR 0) and believed the calculator helped them to identify possible, probable and/or definite cases of ARF (Median 5, IQR 1).

Participants mostly agreed that the diagnosis calculator is quick and accessible to use when evaluating a patient (Median 5, IQR 1) assisted them in determining treatment plans (Median 5, IQR 0), improved their knowledge regarding ARF (Median 5, IQR 1) and improved their confidence (Median 5, IQR 1).

Those who submitted written feedback mainly indicated that the *ARF Diagnosis Calculator* as a usable and easily accessible tool for diagnosis. The majority of respondents nominated ease of use as the main strength of the calculator. Noted weakness comprised the lower utility of the calculator in settings where investigations were unavailable, particularly in remote community settings where blood test results are not rapidly available. In these settings one recommendation was to explicitly state that a low threshold should be used for transfer to and investigation in a tertiary setting to avoid under-diagnosis of ARF.

In addition, some believed the calculator to be overly sensitive without recognising the need to actively exclude alternative diagnoses that mimic ARF. Lastly, although the calculator was considered very easy to use, legislative pop-up notices built into the RHDApp were noted to detract from the experience. A double negative on one-screen was also identified as a potential source for mistakes.

### Semi-structured interviews

Five clinicians experienced in ARF consented to be interviewed. Five overarching themes from the content analysis were identified. These themes were:

- The diagnosis of ARF remains challenging
- The *ARF Diagnosis Calculator* is easily accessible, easy to use, informative and provides educational opportunities for junior staff
- Inclusion of specific medical information is valued
- In remote settings, the *ARF Diagnosis Calculator* is less helpful
- The *ARF Diagnosis Calculator* can be non-specific if the full clinical picture is not appreciated.

A key theme to emerge from interviews was that the diagnosis of ARF is highly challenging, requiring clinical acumen and experience in the heterogeneous nature of ARF presentations. Therefore, while the ARF Diagnosis Calculator helpfully supports diagnosis, expert clinical decision was emphasised as being of most importance rather than an algorithm. Potential oversimplification of a difficult diagnosis through reliance on an app was of concern. Concern was expressed about the chance for missed diagnostic opportunities, particularly where junior medical staff and the locum workforce may lack experience with ARF diagnosis. Participants also noted that the distinction between possible and probable ARF diagnostic categories is subjective, which is problematic since the duration of recommended secondary antibiotic prophylaxis differs greatly between these diagnoses [3]. In these cases, they emphasised the importance of actively considering and investigating for other potential differential diagnoses and seeking expert advice.

While noting inherent clinical challenges in ARF diagnosis, all clinicians indicated that the *ARF Diagnosis Calculator* is easily accessible in point of care settings, easy to use, informative and provides educational opportunities for junior staff. Many agreed that having the calculator on one’s phone, as opposed to hard copy clinical guidelines or electronic guidelines on a desk computer, assisted clinicians to use the diagnostic criteria and identify cases of ARF. Clinicians expressed confidence overall in the use of the *ARF Diagnosis Calculator* by clinical teams in hospital settings, noting the caveats that all investigation results should be taken into account and that all diagnostic assignments made by the calculator should be discussed with senior clinicians. All clinicians agreed that for a transient workforce, which typifies staffing in the Northern Territory, where many clinicians have little experience in ARF, the calculator and RHDApp are very informative and educational.

Having easy access to descriptions of the major and minor ARF diagnostic criteria (Table 1) with definitions available on selecting an information button, was considered very valuable. Users appreciated the availability of a table showing age-specific upper limits of normal for PR Interval (a parameter measured from electrocardiography and a minor Jones criterion), and reference ranges for streptococcal serology (antistreptolysin O or antiDNase B titres). Video footage of Sydenham’s Chorea to help clinicians recognise this ARF manifestation, available on selecting the information button about Sydenham’s Chorea, was noted as a strength.

For remote settings, the experienced clinicians indicated they did not believe the calculator performed as well, given that investigation results that need to be entered are often unavailable. The suggestion was made that missing results should allow the calculator to provide a risk assessment with recommendation for transfer to hospital rather than aiming to confirm a diagnosis promptly. Echocardiogram is essential for diagnostic workup of suspected ARF cases but is only accessible in tertiary centres except during infrequent, scheduled remote clinic visits. In many remote settings, required blood tests results (erythrocyte sedimentation rate, streptococcal serology titres) may be unavailable, or only available with long turnaround times. Consequently, many criteria cannot be entered in the calculator and the RHDApp returns a “Possible ARF” or “No ARF” result.

As noted in survey free-text responses, concerns about the calculator being oversensitive (i.e. inadequately specific) were raised. The experienced clinicians noted that inexperienced users may be unaware that a range of common conditions endemic to the Northern Territory, such as septic arthritis or disseminated gonococcal infection, can fulfil criteria as “Definite ARF” and need to be actively excluded to avoid mistakenly labelling someone as having ARF when there is a confirmed alternative diagnosis. Despite this, the problems of missed diagnoses and missed opportunities for institution of secondary prevention were still recognised as occurring despite the criteria being set for high sensitivity in high-risk populations.

The experienced clinicians complained about there being too many messages to click through to be able to access the calculator. Three boxes—an information page, a disclaimer, and statement that ARF is a legislated notifiable disease in parts of Australia—open sequentially before use of the calculator can commence. Despite the annoyance, most conceded that this was likely required. Another concern was the use of double negatives in “Confirm the patient has NOT had a previous definite diagnosis of ARF or RHD” with answer options “Confirm – no past ARF or RHD” or “No – Did have past ARF or RHD”. These were considered to distract from the calculator’s ease of use.

### Diagnosis calculator versus clinical panel

Thirty-five patients assigned a diagnosis by the expert clinical panel had their demographic and clinical data entered into the *ARF Diagnosis Calculator*. The clinical panel ‘gold standard’ diagnoses are shown in Table 3. Thirty-one (89%) diagnoses obtained using the *ARF Diagnosis Calculator* matched the diagnosis assigned by the clinical panel. The Kappa coefficient was 0.767 (96% CI: 0.568–0.967), consistent with excellent agreement. Sensitivity and specificity were 89% and 96% overall; app accuracy was best for ‘definite ARF’ and ‘no ARF’ (Table 3). Of the four diagnoses that differed, the calculator identified two cases as “definite” cases that were classified as “possible” and “not ARF” by the clinical panel. The first was initially labelled as having monoarthritis (inflammation of a single joint) however on review of the case notes by the clinical panel the case was more in keeping with monoarthralgia (pain of a single joint). This resulted in a “possible ARF” diagnosis from the clinical panel, whereas monoarthritis entered in the *ARF Diagnosis Calculator* had resulted in a diagnosis of “definite ARF”. The second case involved an instance of aseptic (culture-negative) monoarthritis in a high-risk individual with fever, raised C-reactive protein (a blood inflammatory marker) and elevated streptococcal serology (antiDNaseB titre). There were no echocardiogram findings of carditis. This individual however had received antibiotics prior to the joint aspirate being performed, as a consequence of delayed transportation to hospital for the aspiration. The aspirate was turbid in appearance but had no bacterial growth. While the *ARF Diagnosis Calculator* had classified the case as “definite ARF” based on the culture-negative monoarthritis, the clinical context was considered more in keeping with a septic arthritis but with sterilisation of cultures from prior receipt of antibiotics.

**Table 3:** Sensitivity and specificity of the ARF Diagnosis Calculator compared with Gold Standard (expert clinical panel review) applied to patients with suspected rheumatic fever, Royal Darwin Hospital, 2018–2019

	Gold standard diagnostic process	ARF diagnosis calculator
Not ARF (alternative diagnosis)	4	4
Possible ARF	6	5
Probable ARF	2	1
Definite ARF	23	25

	Gold standard positive	Gold standard negative
*ARF classification: definite*		
Positive by calculator	23	2
Negative by calculator	0	10
Sensitivity of calculator = 100% Specificity of calculator = 83%		
*ARF classification: probable*		
Positive by calculator	1	0
Negative by calculator	1	33
Sensitivity = 50% Specificity = 100%		
*ARF classification: possible*		
Positive by calculator	4	1
Negative by calculator	2	28
Sensitivity of calculator = 67%		
Specificity of calculator = 97%		
*ARF Classification: No*		
Positive by calculator	3	1
Negative by calculator	1	30
Sensitivity of calculator = 75% Specificity of calculator = 97%		
*ARF classification: concordance of all options*		
Positive by calculator	31	4
Negative by calculator	4	101
Sensitivity of calculator = 89% Specificity of calculator = 96%		

In the other two instances with discrepant diagnoses, one was initially considered a “possible” diagnosis of ARF however a plausible differential diagnosis of staphylococcal sepsis was confirmed, and the clinical panel assigned the diagnosis as “not ARF”. Using the calculator, the diagnosis was “possible” if the maximum erythrocyte sedimentation rate (a blood inflammatory marker) was used and “not ARF” if the initial erythrocyte sedimentation rate was used. This individual had a previous episode of definite ARF and so was likely investigated more thoroughly with repeat erythrocyte sedimentation rate which resulted in the calculator diagnosis of “possible” ARF. The fourth instance comprised monoarthritis in a high-risk individual with fever and positive streptococcal serology, considered “probable ARF” by the clinical panel and “possible” by the calculator. The different diagnoses obtained arose since in uncertain cases, the calculator requires the user to indicate whether ARF is considered the most likely diagnosis: selecting yes gives a “probable ARF” answer, selecting uncertain (the default used for this exercise) gives “possible ARF” and selecting no gives “no ARF” as the diagnosis.

### Revision of the *ARF Diagnosis Calculator*

Evaluation findings were workshopped with RHDAustralia doctors (A.P.R. and B.J.C) and senior nurse (DM), and sixteen changes were made in response to the study findings, as shown in Additional file 1: Table S1. The revised version of the *ARF Diagnosis Calculator* was finalised in May 2021 and this remains the current embedded version in the RHDApp.

## Discussion

### Principal findings

This study provides evidence for high acceptability, usability and accuracy of the *ARF Diagnosis Calculator* for the diagnosis of ARF. Having accessible guidance and decision support at point of care was identified as a strength, with the app platform being preferred to hardcopy or desktop computer electronic guidelines. Valued features of the calculator included the embedded educational content and specific medical information such as reference ranges for laboratory tests. Several criticisms were made. The number of pop-up messages to be clicked through was considered distracting. Improved wording for clarity was recommended for some sections. The need to actively exclude alternative diagnoses to avoid false-positive ARF diagnoses was considered to need more emphasis. The required information was already provided by the calculator, but was sometimes being overlooked in using the calculator, highlighting that a balance is needed between messages that must be read (needing to be clicked on to move through) versus messages which are shown on the page, but can be lost in the volume of information. Finally, experienced clinicians noted that electronic decision making is not a substitute for clinical experience. The calculator can provide valuable diagnostic assistance, but final assignment of diagnosis for reporting and long-term clinical management purposes should be made by an experienced clinician.

Incorrect assignment of diagnosis occurred in 4/35 (11%) of patients using the calculator compared with an expert clinical panel. Reasons for the discrepancies included that: 1. The calculator does not allow input of results confirming an alternative diagnosis (such as a blood culture result positive for a bacterial pathogen); 2. Nuances from the history or clinical examination could not be appreciated from the summarised data provided for entry into the calculator; 3. Clinical judgement is required to differentiate possible from probable ARF [7]; the clinician needs to draw on their expertise and a broad range of information beyond what is included in the diagnostic criteria (such as age, family history and clinical response to treatment). Interview data also supported this point, reiterating that an electronic decision support tool is not a substitute for expert clinical decision making.

Feedback obtained during this evaluation process was conveyed to the calculator development and review team. Changes were workshopped with RHDAustralia clinicians with sixteen changes made as a result (Additional file 1: Table S1). The number of pop-up messages on opening the app were reduced by combining the disclaimer with agreement to notify ARF in parts of Australia where ARF is a notifiable condition (points 1 and 2, Additional file 1: Table S1). The ‘double negative’ was removed and reworded. It was felt that including results which signify other diagnoses would unnecessarily add to the calculator’s complexity. Instead, strengthened wording was added to state: ‘The Jones Criteria and the diagnosis provided by this calculator assume differential diagnoses have been considered, tested for and excluded. Clinical signs also found in septic arthritis, bacteraemia, lupus and numerous other conditions when entered into the app can result in a diagnosis of possible, probable or even definite ARF being displayed’ (point 7, Additional file 1: Table S1). Challenges differentiating possible form probable ARF were addressed by adding wording to support decision making (point 10, Additional file 1: Table S1).

Difficulties in using the app when results are missing, such as in remote settings, were addressed through revised wording including: ‘If echocardiogram is pending, base decision on clinical assessment until echocardiogram result is available,’ and ‘If results are pending or unavailable, do not select this item, click ‘continue’. (points 12–16, Additional file 1: Table S1). To reinforce the diagnostic complexity of ARF and requirement for specialist input, the following wording was added when the calculator’s diagnosis is displayed: ‘In complicated or early cases, or those with incomplete investigations, seek specialist advice.’ (points 6 and 7, Additional file 1: Table S1).

Oversensitivity and lessened specificity in ARF diagnosis as noted by survey and interview participants, is a product of the current Jones diagnostic criteria for use in high-risk settings (Table 1) on which the calculator is based, rather than being a problem of the *ARF Diagnosis Calculator* per se. Under-diagnosis puts children at risk of missed prescription of long-term antibiotics to prevent ARF recurrences that lead to rheumatic heart disease. From a medical and patient outcome perspective, it is therefore appropriate to set the criteria to achieve high sensitivity at the cost of specificity. This is what serial revisions of the diagnostic criteria have sought to achieve in Australia. Revised wording in the app appearing on the diagnosis page has now been strengthened to convey the message that use of the diagnostic criteria and therefore the *ARF Diagnosis Calculator* assumes that differential diagnoses have been considered, tested for and excluded (point 9, Additional file 1: Table S1).

The use of mHealth to improve systems and diagnostic performance in health care has been increasing. This study provides evidence for the acceptability and usability of an ARF Diagnosis Calculator however emphasises that this should not be at the expense of clinical acumen in decision making. Decision support tools have been both supported and encouraged by clinicians within both emergency department settings and outpatient maternity care in previous studies [11, 12]. In 2021, the replacement of paper-based nutrition assessments of ex-premature infants with an mHealth tool in rural Rwanda, was similarly assessed to improve efficiency and completeness of assessments; however, did not lead to a significant improvement in accuracy, highlighting the importance of clinical assessment apart from mHealth [17]. Nevertheless, this tool was found to be associated with a reduction in stunting, underweight and inadequate interval growth at 6 months corrected age. Our study also supports mHealth as an educational tool and as an easy to access form of medical guidelines. Evidence however has been inconclusive in this area with improvements noted with or without mHealth interventions in compliance to neonatal protocols in Ghana [18]. Similarly in rural India improvements were noted in reducing barriers to healthcare rapport but did not lead to a significant improvement in knowledge [19].

### Limitations

This study is limited in its generalisability since only doctors participated, whereas the RHDApp and *ARF Diagnosis Calculator* are intended for use by a broad range of users including nurses and Aboriginal Health Practitioners. Other users may have provided different feedback. However, participating doctors represented different work settings and levels of seniority, supporting generalisability. Sample sizes were small, but triangulation of data across three methodological approaches revealed similar themes and allowed confirmation of findings, strengthening the study’s internal validity. As a predominantly qualitative analysis, generalisability is not the intent of this study but instead this study has provided evidence for the usability and confidence in mHealth for ARF diagnosis in the Northern Territory. The absence of a diagnostic test for ARF created a challenge for testing accuracy of calculator performance but use of an expert clinical panel provided the best option available.

## Conclusion

Effective dissemination of knowledge about ARF and about resources to support clinicians in diagnosing and managing ARF are a critically important part of the overall strategy required to address morbidity and mortality arising from this condition [20]. The *ARF Diagnosis Calculator* is an informative, usable, acceptable and accurate tool to assist clinicians with ARF diagnosis. Revisions made in response to this evaluation have addressed the critiques and recommendations that end-users and clinical experts provided. ARF remains prevalent yet under-diagnosed. Clinicians lack familiarity with the condition and diagnostic complexity is high. This situation makes electronic decision support tools which are accurate and user-friendly a particularly valuable addition. Further research within mHealth is needed to assess the use of algorithms against traditional clinical assessment to avoid the over-reliance on guidelines.

## Supplementary Information

**Additional file 1**. Summary of revisions to the ARF Diagnosis Calculator in response to evaluation findings.

## Data Availability

The datasets used and/or analysed during the current study are available from the corresponding author on reasonable request.

## Abbreviations

ARF: Acute Rheumatic Fever
RHD: Rheumatic Heart Disease
GAS: Group A β-haemolytic streptococcus bacterium
AHA: American Heart Association
WHO: World Health Organisation
ESR: Erythrocyte Sedimentation Rate
mHealth: Mobile Health
Apps: Mobile applications
COREQ: Consolidated criteria for reporting qualitative research
NT: Northern Territory
WA: Western Australia
SA: South Australia
TEHS: Top End Health Service

## References

[1] Australian Institute of Health and Welfare. Acute rheumatic fever and rheumatic hart disease in Australia, 2015–2019. Cat.no. CVD 90. Canberra: AIHW; 2021. Available from: https://www.aihw.gov.au/getmedia/ed8003f2-f2d6-41fc-ac7e-7ab5189f56ac/Acute-rheumatic-fever-and-rheumatic-heart-disease-in-Australia-2015-2019.pdf.aspx?inline=true.

[2] Ralph AP, Carapetis JR (2013). Group A streptococcal diseases and their global burden. Curr Top Microbiol Immunol.

[3] Ralph AP, Noonan S, Wade V, Currie BJ (2021). The 2020 Australian guideline for prevention, diagnosis and management of acute rheumatic fever and rheumatic heart disease. Med J Aust.

[4] Gewitz MH, Baltimore RS, Tani LY, Sable CA, Shulman ST, Carapetis J (2015). Revision of the Jones Criteria for the diagnosis of acute rheumatic fever in the era of Doppler echocardiography: a scientific statement from the American Heart Association. Circulation.

[5] Jones T (1944). The diagnosis of rheumatic fever. J Am Med Assoc.

[6] Remond M. Informing the prevention, diagnosis and management of acute rheumatic fever and rheumatic heart disease in Aboriginal Australian and Torres Strait Islander populations. [Dissertation]. Townsville: James Cook University; 2014.

[7] RHDAustralia (ARF/RHD writing group). The 2020 Australian guideline for prevention, diagnosis and management of acute rheumatic fever and rheumatic heart disease (3rd edition) Darwin: Menzies School of Health Research; 2020. Available from: https://www.rhdaustralia.org.au/arf-rhd-guideline

[8] Wakerman J, Humphreys J, Bourke L, Dunbar T, Jones M, Carey TA, et al. Assessing the Impact and Cost of Short-Term Health Workforce in Remote Indigenous Communities in Australia: A Mixed Methods Study Protocol. JMIR Res Protoc. 2016;5(4):e135. 10.2196/resprot.5831 PMC5067357 27697750

[9] Hardie K, Ralph AP, de Dassel JL (2020). RHD elimination: action needed beyond secondary prophylaxis. Aust N Z J Public Health.

[10] Paradis M, Stiell I, Atkinson KM, Guerinet J, Sequeira Y, Salter L, et al. Acceptability of a mobile clinical decision tool among emergency department clinicians: development and evaluation of the Ottawa rules app. JMIR mHealth and uHealth. 2018;6(6):e10263. 10.2196/10263 PMC6018230 29891469

[11] Quan AML, Stiell I, Perry JJ, Paradis M, Brown E, Gignac J (2020). Mobile clinical decision tools among emergency department clinicians: web-based survey and analytic data for evaluation of the ottawa rules app. JMIR Mhealth Uhealth.

[12] Waldemar V, Ademir Luiz Do P, Yusra A-L, Adriana T, Fabiana Santos P, Dayane A, et al. d-GDM: a mobile diagnostic decision support system for gestational diabetes. Arch Endocrinol Metab. 2019;63(5):524–30. doi:10.20945/2359-3997000000171. 10.20945/2359-3997000000171 PMC10522262 31482958

[13] World Health Organisation (WHO). mHealth: New horizons for health through mobile technologies: second global survey on eHealth. Geneva, Switzerland: WHO; 2011.

[14] RHDAustralia. ARF & RHD Guideline app - with ARF diagnosis calculator: iPhone. 2014. Available from: https://www.rhdaustralia.org.au/apps.

[15] Creswell JW, Poth CN. Qualitative inquiry & research design. Choosing among five approaches (4th Edition). California: SAGE Publications; 2018.

[16] Telethon Kids Institute. START Study (Searching for a technology-driven acute rheumatic fever test). Available from: www.telethonkids.org.au/projects/working-towards-a-better-understanding-of-arf. 10.1136/bmjopen-2021-053720 PMC8444258 34526345

[17] Nemerimana M, Karambizi AC, Umutoniwase S, Barnhart DA, Beck K, Bihibindi VK, et al. Evaluation of an mHealth tool to improve nutritional assessment among infants under 6 months in paediatric development clinics in rural Rwanda: Quasi‐experimental study. Matern Child Nutr. 2021;17(4):e13201 10.1111/mcn.13201 PMC8476404 33960693

[18] Amoakoh HB, Klipstein-Grobusch K, Agyepong IA, Amoakoh-Coleman M, Kayode GA, Reitsma JB (2020). Can an mhealth clinical decision-making support system improve adherence to neonatal healthcare protocols in a low-resource setting?. BMC Pediatr.

[19] Charanthimath U, Katageri G, Kinshella M-LW, Mallapur A, Goudar S, Ramadurg U, et al. Community health worker evaluation of implementing an mHealth application to support maternal health care in Rural India. Front Glob Women's Health. 2021;2:645690. 10.3389/fgwh.2021.645690 PMC8593958 34816198

[20] Wyber R, Noonan K, Halkon C, Enkel S, Cannon J, Haynes E (2020). Ending rheumatic heart disease in Australia: the evidence for a new approach. Med J Aust.

