# Supplementary material for: Evaluation of an ARF diagnosis calculator: a survey and content analysis
Source: BMC Med Inform Decis Mak. 2022 Mar 26;22:77. doi: 10.1186/s12911-022-01816-7 (PMC8961951; doi:10.1186/s12911-022-01816-7)
Supplement: Supplementary file 1 — Additional file 1. Summary of revisions to the ARF Diagnosis Calculator in response to evaluation findings. [file 12911_2022_1816_MOESM1_ESM.docx]

Supplementary Table 1: Revisions to the ARF Diagnosis Calculator in response to evaluation findings

| Location | Current Text 22/6/2020 | Revised text (response to evaluation) |
| --- | --- | --- |
| 1. Do you agree? | By clicking 'YES' you agree to the terms outlined in the previous disclaimer and that the user of the application possesses the appropriate clinical judgement and assessment skills to make informed clinical decisions and will only use the results from this tool as an aid to support ARF diagnosis. | Do you agree?  In Australia, ARF is notifiable in QLD, NT, WA, SA and NSW, where cases MUST be notified to the local Public Health Unit and the regional ARF/RHD control programs.  By clicking 'YES' you agree to the terms outlined in the disclaimer and that the user of the application possesses the appropriate clinical judgement and assessment skills to make informed clinical decisions. Results from the use of this tool will act as an aid to support ARF diagnosis. |
| 2. Notifying ARF | ARF is a legislated notifiable disease in some countries. In Australia, it is notifiable in QLD, NT, WA, SA and NSW, where cases MUST be notified to the local Public Health Unit and the regional ARF/RHD control programs. |  |
| 3. Confirm the patient has NOT had a previous definite diagnosis of ARF or RHD | Confirm the patient has NOT had a previous definite diagnosis of ARF or RHD | Confirm the risk level |
| 4. Confirm previous definite: Option: CONFIRM - No past ARF or RHD | (Green button) CONFIRM - No past ARF or RHD | (Green button) No past ARF or RHD |
| 5. Confirm previous definite: Option: NO- DID have past ARF or RHD | (Red button) NO - DID have past ARF or RHD | (Red button) Past ARF or RHD |
| 6. Result: NO ARF | In complicated cases, seek expert advice before categorically excluding ARF as a diagnosis.  If you require further information please contact your state / territory RHD control program or local public health departments.  Contact numbers: …… | In complicated or early cases, or those with incomplete investigations, seek specialist advice before categorically excluding ARF as a diagnosis.  If you require further information, please contact your state / territory RHD control program or local public health departments.  Contact numbers: …… |
| 7. Result: Possible ARF (uncertain) | ARF is a legislated notifiable disease in some countries. In Australia, it is notifiable in QLD, NT, WA, SA and NSW, where **POSSIBLE ARF (Uncertain) cases MUST be notified to the regional ARF/RHD control program.**  People suspected to have ARF may not fulfil the criteria for a number of reasons including atypical presentation, delayed presentation, and incomplete initial assessment. Such people may fit into the category of 'probable ARF'.  Further investigation may be required, such as streptococcal serology and detailed medical history.  **People with possible ARF should receive 12 months of secondary prophylaxis and be referred for specialist reassessment (including echocardiography) at that time. If there has been no evidence of recurrent ARF, and no evidence of heart valve damage on echocardiography, the specialist may consider ceasing secondary prophylaxis. Such people should be educated to monitor future sore throats and identify symptoms of potential ARF and seek prompt treatment.**  Contact numbers………. | In complicated or early cases, or those with incomplete investigations, seek specialist advice.  The Jones Criteria and the diagnosis provided by this calculator assume differential diagnoses have been considered, tested for and excluded. Clinical signs of septic arthritis, bacteraemia, lupus and numerous other conditions when entered into the app can result in a diagnosis of possible, probable or even definite ARF being displayed.  People suspected to have ARF may not fulfil the criteria for many reasons including atypical or delayed presentation, incomplete initial assessment or investigations. Such people may fit into the category of 'possible’ or ‘probable’ ARF', and specialist advice is required.  Further investigation may be required, such as streptococcal serology and detailed medical history.  People with possible and probable ARF should begin secondary prophylaxis and be referred for specialist assessment (including follow up echocardiography). See Table 10.2 on page 184 of the [guidelines](https://rhdaustralia.opcentral.com.au/People%20with%20possible%20and%20probable%20ARF%20should%20begin%20secondary%20prophylaxis%20and%20be%20referred%20for%20specialist%20assessment%20(including%20follow%20up%20echocardiography).%20See%20Table%2010.2%20(provide%20hyperlink)%20for%20recommended%20follow%20up%20and%20duration%20of%20secondary%20prophylaxis%20for%20possible%20and%20probable%20ARF.) for recommended follow up and duration of secondary prophylaxis for possible and probable ARF.  **POSSIBLE and PROBABLE ARF cases must also be reported to the regional ARF/RHD control program.**  If you require further information, please contact your state / territory RHD control program or local public health departments.  Contact numbers………. |
| 8. Result: Definite ARF | Diagnosis  Definite ARF  ARF is a legislated notifiable disease in some countries. In Australia, it is notifiable in QLD, NT, WA, SA and NSW, where cases MUST be notified to the local Public Health Unit and the regional ARF/RHD control programs.  If you require further information please contact your state / territory RHD control program or local public health departments.  Contact numbers……. | Diagnosis  Definite ARF  It is recommended that you seek specialist advice and arrange admission to hospital, even if this is a recurrent episode of ARF.  The Jones Criteria and the diagnosis provided by this calculator assume differential diagnoses have been considered, tested for and excluded. Clinical signs of septic arthritis, bacteraemia, lupus and numerous other conditions when entered into the app can result in a diagnosis of possible, probable or even definite ARF being displayed.  In Australia, ARF is notifiable in QLD, NT, WA, SA and NSW, where cases MUST be notified to the local Public Health Unit and reported to the regional ARF/RHD control programs.  See Table 10.2 on page 184 of the [guidelines](https://rhdaustralia.opcentral.com.au/People%20with%20possible%20and%20probable%20ARF%20should%20begin%20secondary%20prophylaxis%20and%20be%20referred%20for%20specialist%20assessment%20(including%20follow%20up%20echocardiography).%20See%20Table%2010.2%20(provide%20hyperlink)%20for%20recommended%20follow%20up%20and%20duration%20of%20secondary%20prophylaxis%20for%20possible%20and%20probable%20ARF.) for recommended follow up and duration of secondary prophylaxis for possible and probable ARF.  <https://www.rhdaustralia.org.au/system/files/fileuploads/arf_rhd_guidelines_3rd_edition_web.pdf#page=184>  **for recommended follow up and duration of secondary prophylaxis.** |
| 9. Probable ARF | Diagnosis  Probable ARF (Highly Suspected)  ARF is a legislated notifiable disease in some countries. In Australia, it is notifiable in QLD, NT, WA, SA and NSW, where **PROBABLE ARF (Highly Suspected) cases MUST be notified to the regional ARF/RHD control program.**  People suspected to have ARF may not fulfil the criteria for a number of reasons including atypical presentation, delayed presentation, and incomplete initial assessment. Such people may fit into the category of 'probable ARF'.  Probable ARF (highly suspected) is defined as a clinical presentation that falls short by either one major or one minor manifestation or the absence of supporting streptococcal serology results, but one in which ARF is considered the most likely diagnosis.  **People with probable ARF (highly suspected) should be managed as for definite ARF under the care of a medical specialist.**  Contact numbers……. | Diagnosis  Probable ARF (Highly Suspected)  People suspected to have ARF may not fulfil the criteria for many reasons including atypical or delayed presentation, incomplete initial assessment or investigations. Such people may fit into the category of 'probable ARF', and specialist advice is required.  The Jones Criteria and the diagnosis provided by this calculator assume differential diagnoses have been considered, tested for and excluded. Clinical signs of septic arthritis, bacteraemia, lupus and numerous other conditions when entered into the app can result in a diagnosis of possible, probable or even definite ARF being displayed.  Probable ARF (highly suspected) is defined as a clinical presentation that falls short by either one major or one minor manifestation or the absence of supporting streptococcal serology results, but one in which ARF is considered the most likely diagnosis.  ARF is notifiable in QLD, NT, WA, SA and NSW, where POSSIBLE and **PROBABLE ARF cases MUST also be notified to the regional ARF/RHD control program.**  **People with possible and probable ARF should begin secondary prophylaxis and be referred for specialist assessment (including follow up echocardiography). See Table 10.2 (provide hyperlink) for recommended follow up and duration of secondary prophylaxis for possible and probable ARF.**  If you require further information please contact your state / territory RHD control program or local public health departments.  Contact numbers……. |
| 10. Is ARF considered to be the most likely diagnosis? | Is ARF considered to be the most likely diagnosis? | Is ARF considered to be the most likely diagnosis?  Consider:   - Family history - Living circumstances - Age - Sex - Joint pain   ‘info’ button text:  In making this decision, consider the person’s:  Family history (a family history of ARF or RHD substantially increases an individual’s risk)  Living circumstances (crowded or low socioeconomic conditions increase the risk)  Age (children aged between 5-14years are most likely to get ARF)  Sex (females are more likely to get ARF)  If joint pain is present, the response to NSAIDS (ARF joint pain usually decreases substantially after commencing NSAIDS including aspirin and naproxen). It is safer to consider ARF than ascribe joint symptoms to musculoskeletal injury, especially if not clear how the injury occurred, or if fever is present. |
| 11. Major Manifestation (High Risk): Carditis | Rheumatic carditis refers to the active inflammation of the myocardium, endocardium and pericardium that occurs in ARF. The most common manifestation is the involvement of the endocardium presenting as a valvulitis, especially of the mitral and aortic valves. The presence of carditis can often be found at initial presentation, but occasionally signs of carditis appear later, and usually within the first 2-6 weeks. | Rheumatic carditis refers to the active inflammation of the myocardium, endocardium and pericardium that occurs in ARF.  Echocardiogram confirms the diagnosis of acute carditis, which may be present in the absence of a murmur. If echocardiogram is pending, base decision on clinical assessment until echocardiogram result is available.  The most common manifestation is the involvement of the endocardium presenting as a valvulitis, especially of the mitral and aortic valves. The presence of carditis can often be found at initial presentation, but occasionally signs of carditis appear later, and usually within the first 2-6 weeks. |
| 12. Major Manifestation (Low Risk): Carditis | Rheumatic carditis refers to the active inflammation of the myocardium, endocardium and pericardium that occurs in ARF. The most common manifestation is the involvement of the endocardium presenting as a valvulitis, especially of the mitral and aortic valves. The presence of carditis can often be found at initial presentation, but occasionally signs of carditis appear later, and usually within the first 2-6 weeks.  There are four specific findings including………… | Rheumatic carditis refers to the active inflammation of the myocardium, endocardium and pericardium that occurs in ARF.  Echocardiogram confirms the diagnosis of acute carditis, which may be present in the absence of a murmur. If echocardiogram is pending, base decision on clinical assessment until echocardiogram result is available.  The most common manifestation is the involvement of the endocardium presenting as a valvulitis, especially of the mitral and aortic valves. The presence of carditis can often be found at initial presentation, but occasionally signs of carditis appear later, and usually within the first 2-6 weeks.  There are four specific findings including………… |
| 13. Minor Manifestation (High Risk): ESR>=30mm/hr OR CRP>=30mg/L | Typically, people with ARF show markers of inflammation. In ARF these include an erythrocyte sedimentation rate equal to or greater than 30 mm/h (high risk populations) or a C-reactive protein (CRP) level equal to or greater than 30 mg/L.  The CRP concentration rises and falls more rapidly than the ESR. The ESR may remain elevated for 3–6 months. | If results are pending or unavailable, do not select this item, click ‘continue’.  Typically, people with ARF show markers of inflammation. In ARF these include an erythrocyte sedimentation rate equal to or greater than 30 mm/h (high risk populations) or a C-reactive protein (CRP) level equal to or greater than 30 mg/L.  The CRP concentration rises and falls more rapidly than the ESR. The ESR may remain elevated for 3–6 months. |
| 14. Minor Manifestation (High Risk): Prolonged P-R interval for age on ECG | Some healthy people show prolonged P-R interval, however a prolonged P-R interval that resolves over days or weeks may be a useful diagnostic feature suggesting carditis. If a prolonged P-R interval is detected and ARF is suspected, the ECG should be repeated after 2 weeks, and if still abnormal, it should be repeated again at 2 months to document a return to normal. If it has returned to normal, ARF becomes a more likely diagnosis. | If results are pending or unavailable, do not select this item, click ‘continue’.  Some healthy people show prolonged P-R interval, however a prolonged P-R interval that resolves over days or weeks may be a useful diagnostic feature suggesting carditis. If a prolonged P-R interval is detected and ARF is suspected, the ECG should be repeated after 2 weeks, and if still abnormal, it should be repeated again at 2 months to document a return to normal. If it has returned to normal, ARF becomes a more likely diagnosis. |
| 15. Minor Manifestation (Low Risk): ESR ≥ 60 mm/h OR\CRP ≥ 30 mg/L | Typically, people with ARF show markers of inflammation. In ARF these include an erythrocyte sedimentation rate equal to or greater than 60 mm/h or a C-reactive protein (CRP) level equal to or greater than 30 mg/L.  The CRP concentration rises and falls more rapidly than the ESR. The ESR may remain elevated for 3–6 months. | If results are pending or unavailable, do not select this item, click ‘continue’.  Typically, people with ARF show markers of inflammation. In ARF these include an erythrocyte sedimentation rate equal to or greater than 60 mm/h or a C-reactive protein (CRP) level equal to or greater than 30 mg/L.  The CRP concentration rises and falls more rapidly than the ESR. The ESR may remain elevated for 3–6 months. |
| 16. Minor Manifestation (Low Risk): Prolonged P-R interval for age on ECG | Some healthy people show prolonged P-R interval, however a prolonged P-R interval that resolves over days or weeks may be a useful diagnostic feature suggesting carditis. If a prolonged P-R interval is detected and ARF is suspected, the ECG should be repeated after 2 weeks, and if still abnormal, it should be repeated again at 2 months to document a return to normal. If it has returned to normal, ARF becomes a more likely diagnosis.  …….. | If results are pending or unavailable, do not select this item, click ‘continue’.  Some healthy people show prolonged P-R interval, however a prolonged P-R interval that resolves over days or weeks may be a useful diagnostic feature suggesting carditis. If a prolonged P-R interval is detected and ARF is suspected, the ECG should be repeated after 2 weeks, and if still abnormal, it should be repeated at 2 months to document a return to normal. If it has returned to normal, ARF becomes a more likely diagnosis. |
